# Construction of hybrid regulated mother-specific yeast promoters for inducible differential gene expression

**DOI:** 10.1371/journal.pone.0194588

**Published:** 2018-03-22

**Authors:** Georgios Pothoulakis, Tom Ellis

**Affiliations:** 1 Centre for Synthetic Biology and Innovation, Imperial College London, London, United Kingdom; 2 Department of Bioengineering, Imperial College London, London, United Kingdom; Universite Paris-Sud, FRANCE

## Abstract

Engineered promoters with predefined regulation are a key tool for synthetic biology that enable expression on demand and provide the logic for genetic circuits. To expand the availability of synthetic biology tools for *S*. *cerevisiae* yeast, we here used hybrid promoter engineering to construct tightly-controlled, externally-inducible promoters that only express in haploid mother cells that have contributed a daughter cell to the population. This is achieved by combining elements from the native *HO* promoter and from a TetR-repressible synthetic promoter, with the performance of these promoters characterized by both flow cytometry and microfluidics-based fluorescence microscopy. These new engineered promoters are provided as an enabling tool for future synthetic biology applications that seek to exploit differentiation within a yeast population.

## Introduction

Synthetic biology seeks to expand on existing biotechnology principles and develop technologies that simplify the design and construction of engineered biological devices and systems that carry out tasks [1, 2]. As a result, synthetic biology is defined by its capability to develop novel tools that enable new gene expression control mechanisms. *Saccharomyces cerevisiae* yeast has been one of the most popular chassis for synthetic biology studies as it is a well-studied model organism that enables many applications in biotechnology (e.g. production of complex molecules, traditional and novel biofuels, recombinant proteins) [3–6].

**Fig 1:**
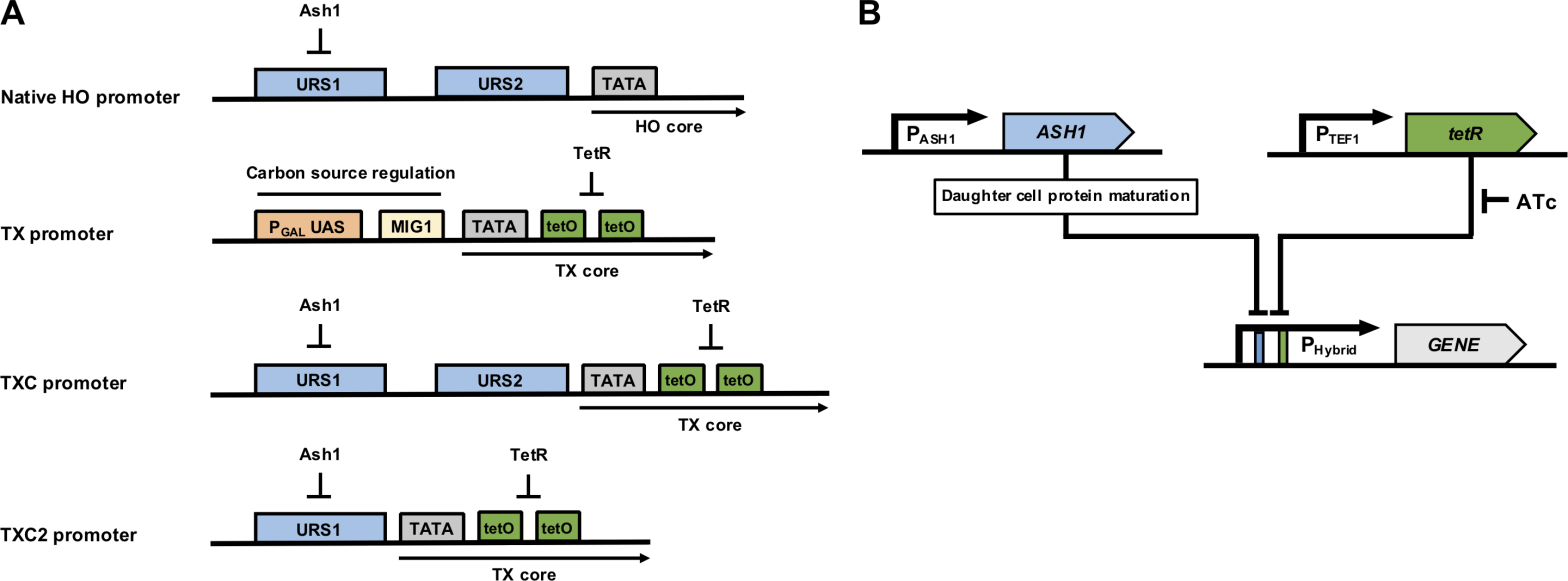
Hybrid promoter design and regulation. (A) Structure schematics of the native *HO* promoter and the synthetic *TX*, *TXC and TXC2* promoters. Regulatory elements are shown as colored rectangular boxes. The *HO* promoter is regulated by two upstream regulatory sites (URS1 and URS2) and is repressed by the Ash1 protein which binds primarily to several sites of the URS1. The *TX* promoter is primarily regulated by the P_GAL_ upstream activation site (UAS) and a Mig1 repressor protein binding site as well as tandem TetR operator sequences. The *TXC* and *TXC2* are *TX*-based hybrid promoters containing either both the URS1 and URS2 or just the URS1 region respectively, upstream of the core *TX* promoter. (B) Diagram of the regulatory network controlling hybrid promoter activation. Hybrid promoter regulatory elements are shown as colored rectangular boxes. Genes are shown as colored arrows. Anhydrous tetracycline (ATc) is inhibiting tetR repression. Ash1 protein is only present in daughter cells.

The availability of well-characterized gene expression control tools is essential for all potential synthetic biology applications. The most popular target for gene expression control in yeast synthetic biology has always been via promoter regulation, as control of transcription is best understood in eukaryotes. Initially, research focused on the characterization and use of native constitutive and inducible promoters, such as the constitutive TEF1, the GDP and the ADH1 promoters, and inducible FIG1 and GAL promoters [7, 8]. In the last decade work has quickly moved to the creation of synthetic promoters that expand beyond the limited regulation options of native promoters. Most synthetic promoters have been developed by mutating natural promoters and/or by adding new operator sites to existing promoters to change their regulation by either controllable repression or activation of transcription [7]. For example, in order to study transcriptional noise, Murphy *et al*. created a series of repressible promoters by taking the native galactose-inducible *GAL1* promoter and adding tetO operator sites to the core promoter region downstream of the TATA-box thus creating a synthetic promoter that is also regulated by TetR [9]. This strategy was further used to create a library of *GAL1*-based TetR-repressible promoters of various strengths and a similar *GAL1*-based LacI-repressible promoter Iibrary inducible via addition of IPTG [10]. Addition of tetO operator sites into the core region of a different yeast promoter (*PFY1* promoter) was also demonstrated to convert this normally constitutive promoter into an externally-inducible one [11].

While these efforts focused on engineering orthogonal regulation into the core of yeast promoters, others have instead looked at engineering regulation by changing the upstream sites that activate expression. Regulated promoters found naturally in the yeast genome typically have upstream enhancer elements that control the rate of transcription in response to inputs, while the core promoter elements serve more to determine the start site and direction of transcription [12]. Recently, a hybrid promoter strategy has come to the fore that takes these two regions from different yeast promoters and matches them together in new combinations. This enables the creation of promoters with enhanced activity or with alternative regulation, and it was initially used as a way to identify and characterize the function of regions in natural promoters. For example, the construction of a *GAL10-CYC1* hybrid promoter helped identify the role of the *GAL4* regulatory region upstream of the *GAL10* gene [13]. However, hybrid promoter engineering is now at the forefront of yeast synthetic biology efforts, thanks to recent work by the Alper lab in both identifying important regulatory and core promoter sequence motifs and using these to realize synthetic promoters with desired characteristics [14–17]. Indeed, by combining the upstream activating sequences (UAS) from different *S*. *cerevisiae* promoters with the core elements of others, Blazeck *et al*. were able to make new promoters stronger than any seen naturally in yeast and to change the regulation of different existing promoters [8]. This work was further expanded by Teo and Chang, who achieved the construction of synthetic hybrid promoters that act as AND-gate controllers by combining inducible upstream activating sequences with an inducible GAL1-based core to create promoters that require two separate conditions to be met in order to activate [12].

So far, all promoter engineering in yeast has focused on constructing promoters to respond to external inputs or internal metabolites and give a reliable, uniform output across the population of cells. However, some natural yeast promoters respond differentially within a population of growing yeast, expressing in mother cells but not in newly-budded daughter cells, or *vice versa*. The *HO* promoter is a classic example of this kind of promoter and the *HO* gene it expresses encodes an endonuclease that causes mating-type switching between *MATa* and *MATα* cells [18, 19]. It is intended to only be active in haploid mother cells so that mating-type switching is only seen in these cells and not in daughter cells or diploid cells [20, 21].

In order to expand the toolset of yeast synthetic biology, so that differential regulation can be controlled within a population of *S*. *cerevisiae* cells, we here set out to engineer hybrid promoters that combine the mother-specific expression of the *HO* promoter with externally-inducible control. The goal was to create promoters that can enable genes of interest, such as cytosolic enzymes, to be activated when desired but restrict their expression to mother cells within the population. Here we show that this can be achieved by the construction of synthetic hybrid promoters that combine core elements from TetR-regulated GAL1-based promoters and upstream elements of the *HO* promoter.

## Results

To produce externally-inducible mother-specific promoters, we employed the hybrid promoter engineering strategy to combine the core promoter region of a synthetic repressible promoter with native upstream regulatory sites known to be bound differently in mother cells versus daughter cells. For the core promoter region, we took sequence from the TetR-regulated *TX* promoter, while for the mother-specific regulatory region, we took upstream sequence from the native *HO* promoter [10, 21].

The *HO* promoter is a tightly-regulated, relatively large promoter that is bound by several activators and repressors in order to control transcription to only occur in that right type of the cell and at specific points during the cell cycle [20–22]. The promoter contains two upstream regulatory regions termed URS1 and URS2, which confine expression to mother cells and to late G1 cell cycle phase [21, 23]. The promoter’s unique mother cell specificity is attributed to the Ash1 negative regulator protein which localizes asymmetrically to daughter cells during budding, and represses *HO* transcription [24]. In the daughter cell, the Ash1 protein binds to specific consensus sequences found in both URS1 and URS2 of the *HO* promoter and prevents its expression [25]. Deletion of URS2, which contains binding sites for a transcriptional activator (SBF) required for activator signal propagation and proper HO promoter activation, does not appear to affect steady-state expression levels of the *HO* gene, but it does shift *HO* expression from late G1 to the anaphase stage, leads to loss of the mother cell specificity and results in almost a third of the daughter cells to switch mating types [23, 24, 26–28]. On the other hand, deletion of URS1 leads to drastically reduced transcription [23].

The *TX* promoter is an engineered variant of the extensively-used *GAL1* promoter, designed to include a tandem pair of *TET* operator sites immediately downstream of its TATA-box in order to enable repression of transcription in the presence of constitutively expressed TetR (**Fig 1A**) [10]. It has an upstream PGAL UAS that confers activation of expression in galactose media, and is also repressed by glucose via a Mig1 binding site just upstream of the TATA-box [10, 29, 30]. The effect of this promoter on GFP expression has been extensively characterised in yeast at the population and single-cell level by flow cytometry, fluorescence microscopy and microfluidic techniques in four previous studies that focused on synthetic biology circuits and on studying regulated gene expression noise. In these published works it has been shown that TetR provides tight repression of the TX promoter in the absence of anhydrous tetracycline (ATc) inducer, and that after induction with a high concentration of ATc, the promoter drives strong expression in all cells in a growing population [10, 31–33].

The *HO* promoter also has a distinct core promoter which is paired with the two upstream regulatory regions, URS1 and URS2, which ensure HO expression is only in haploid mother cells [25]. In contrast to the TX promoter, previous work characterising GFP expression from this promoter by microscopy has shown that it almost only ever expresses in mother cells, and is not active in the daughter cells of a growing population of yeast [28]. The standard HO promoter mother-cell specificity is very high, with only 4.3% of daughter cells expressing any GFP.

For the hybrid promoters constructed here, all sequence upstream of the *TX* promoter TATA-box was removed and replaced with equivalent *HO* promoter upstream sequence. This meant that the PGAL upstream activation sequence (UAS) and the Mig1 repressor sites were both removed, eliminating galactose/glucose regulation. This is particularly important since galactose-induced expression of the *TX* promoter is very strong and would likely mask activation via URS1 or URS2.

Two different configurations of *HO-TX* hybrid promoters were designed, constructed and tested. The first configuration *(TXC)* added both HO promoter regulatory sites (URS1 and URS2) upstream of the TX promoter core, whereas the second *(TXC2)* added only the URS1 site. Both promoters were designed to lack galactose-based regulation but maintain the ability to be repressed by constitutive expression of TetR, which can itself be blocked by addition of ATc or doxycycline to the culture media (**Fig 1A and 1B**). To test these promoters for their expression output, their regulation and cell-cycle performance, they were both placed upstream of a gene encoding a fast-degrading Green Fluorescent Protein (*FD-GFP*). This GFP variant has a fused N-terminal ubiquitin tag that promotes fast degradation in the *S*. *cerevisiae* cytoplasm via deubiquitination, allowing expression dynamics to be monitored over time [34, 35]. This compares to a previously-developed unstable GFP protein designed to shuttle to the nucleus which has been used to characterize the activity of the natural HO promoter in a past study [36]. The promoter-gene combinations were cloned on integrating plasmids and transformed in haploid BY4741 yeast cells (Y02569 strain). For TetR repression, the *tetR* gene under the control of a *TEF1* promoter was also included in both plasmid constructs.

**Fig 2:**
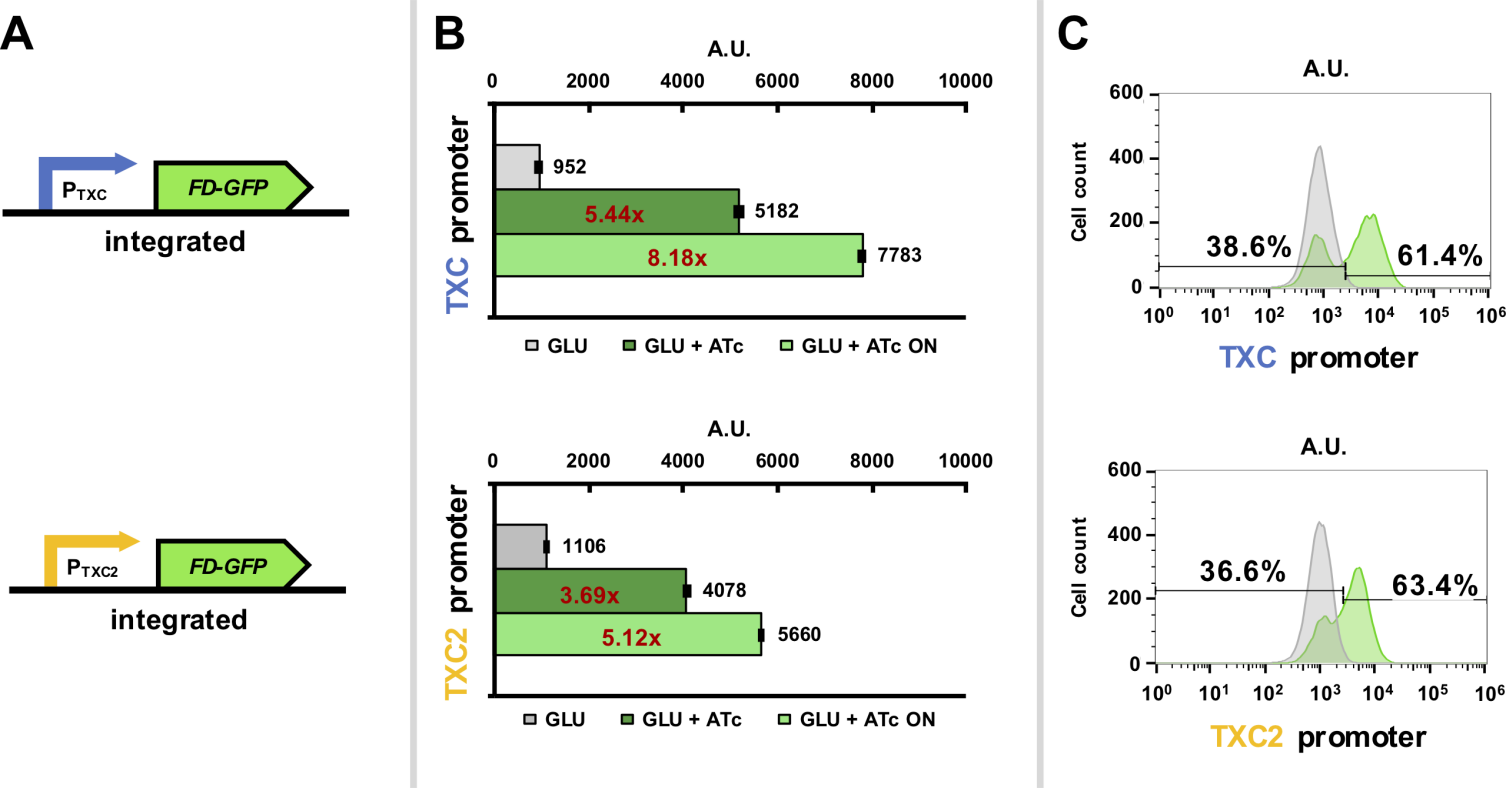
Characterization of genome-integrated hybrid promoter constructs in glucose media using flow cytometry acquisition. (A) Diagrams of the *TXC* and the *TXC2* hybrid promoters driving *FD-GFP* expression. (B) Green fluorescence data from *S*. *cerevisiae* cells expressing the pGPY022 (*TXC* hybrid promoter) and pGPY020 (*TXC2* hybrid promoter) integrated constructs in synthetic complete glucose media after induction with 200 ng/μl anhydrous tetracycline (ATc) for 5 hours. Uninduced samples where no ATc was added were also included. Data show average mean fluorescence values of biological triplicates as determined by flow cytometry. Mean fluorescence values of the “ON” cell populations (cells that are considered to fluoresce), which are determined by the 98th percentile of the uninduced samples, are also shown. The ratios between induced and uninduced cells are shown in red. (C) Histograms of fluorescence intensity over cell count of one of the triplicates of each strain to showcase potential bimodality. The gates that separate the “ON” and “OFF” populations for each sample are also shown along with percentages.

To characterize expression from the constructed promoters, the two strains carrying the new hybrid promoters with the *FD-GFP* gene were first assessed using flow cytometry. Expression from both promoters was induced by relieving TetR-repression by addition of ATc to the glucose-based growth media. As the PGAL UAS and Mig1 sites are not present in the *TXC* and *TXC2* promoters, their expression should not be regulated by carbon source. Green fluorescence per cell was measured 5 hours post-ATc induction for a minimum of ten thousand cells. For both yeast strains the distribution of fluorescence per cell was plotted and the overall mean fluorescence of the population was calculated (**Fig 2**). This revealed that strains with the *TXC* and *TXC2* promoters exhibit minimal expression in glucose media, but expression is activated when ATc is added with fluorescence increasing by 8.18x for the TXC promoter and 5.12x for the TXC2 promoter, when only the “ON” populations are considered. The bimodal distribution is an indication of differential expression in the population, which we attribute to differences in expression between mother and daughter cells, assuming that no spontaneous mutations in the have arisen in the integrated constructs. When the cells analysed from our sample are gated into “ON” and “OFF” populations (OFF defined by the fluorescence range for the 98th percentile of the uninduced sample), we see approximately 40% of the population in the “OFF” state, and the remaining 60% “ON”, albeit at different levels of expression. This is close to the ratio one would expect to see for a growing population of yeast cells expressing with the standard HO promoter, whose differential expression has been shown to be strict, with around 98% of the mother cells expressing from this promoter and 98% of daughter cells not showing any expression [36]. This would result in expression from approximately 60% of cells in a growing population of yeast grown in rich culture media as this is the percentage of mother cells that would expected to be seen at fast growth rates [37]. The ratio is not 50/50, since daughter cells take time to reach a critical size before moving to the S phase, and thus have a longer cell-cycle.

**Fig 3:**
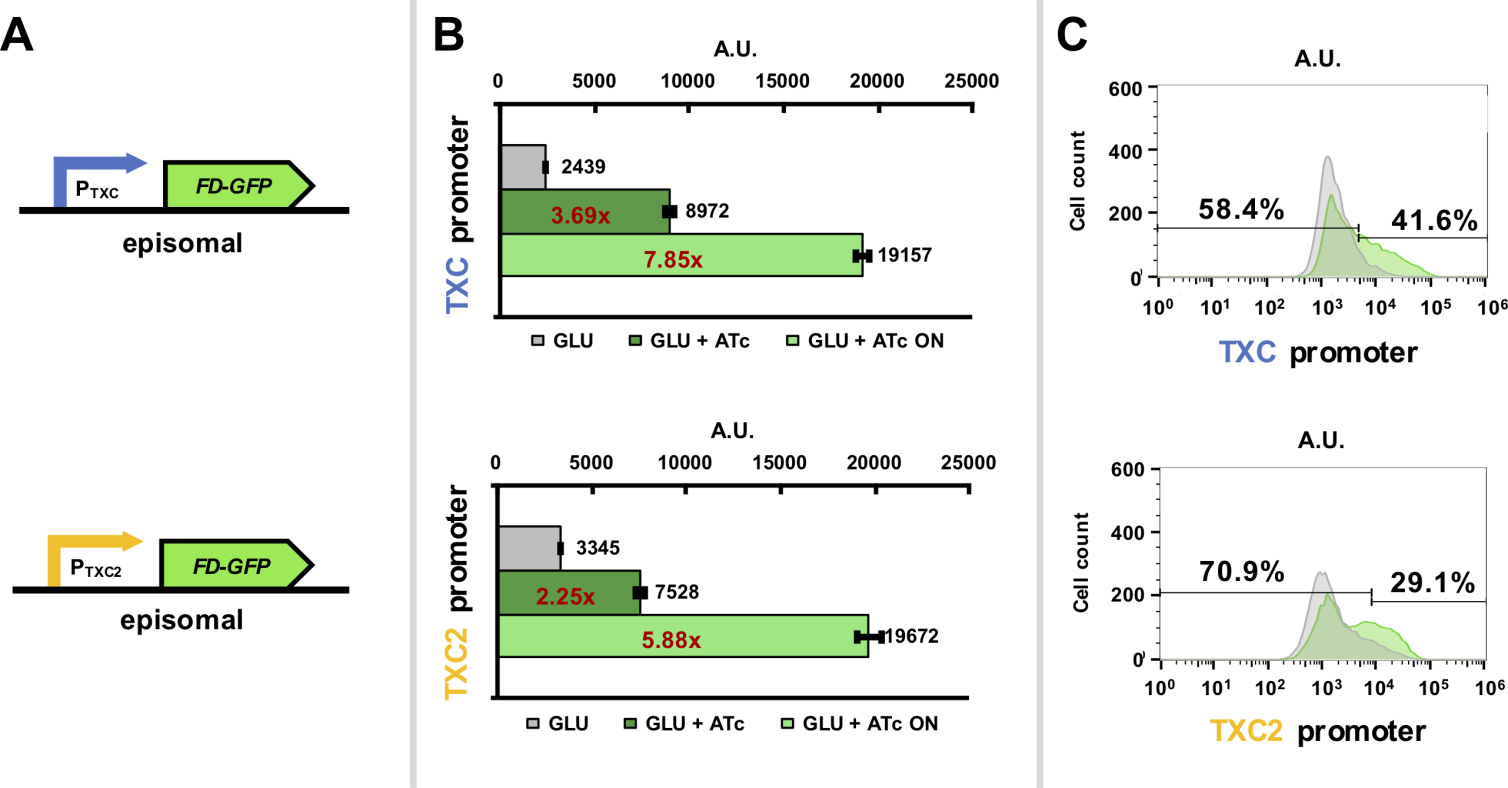
Characterization of hybrid promoter constructs expressed from 2-micron plasmids. (A) Diagrams of the *TXC* and the *TXC2* hybrid promoters driving *FD-GFP* expression. (B) Green fluorescence data from *S*. *cerevisiae* cells expressing the pGPY055 (*TXC* hybrid promoter) and pGPY054 (*TXC2* hybrid promoter) integrated constructs in synthetic complete glucose media after induction with 200 ng/μl anhydrous tetracycline (ATc) for 5 hours. Uninduced samples where no ATc was added were also included. Data show average mean fluorescence values of biological triplicates as determined by flow cytometry. Mean fluorescence values of the “ON” cell populations (cells that are considered to fluoresce), which are determined by the 98th percentile of the uninduced samples, are also shown. The ratios between induced and uninduced cells are shown in red. (C) Histograms of fluorescence intensity over cell count of one of the triplicates of each strain to showcase potential bimodality. The gates that separate the “ON” and “OFF” populations for each sample are also shown along with percentages.

Both hybrid promoters were also assessed for their expression when placed on 2-micron plasmids, rather than when integrated into the yeast genome. As these are high-copy plasmids (usually 40–60 copies per haploid cell) they have the potential to greatly increase overall protein expression from the hybrid promoters [38]. Flow cytometry fluorescence characterization of strains containing these plasmids showed some evidence for bimodal expression and also revealed an expected increase in *FD-GFP* expression per cell (**Fig 3;** 2.5-fold for the TXC promoter and 3.5-fold for the TXC2 promoter over the integrated version when only the “ON” cell populations are considered). While this represents increased expression per cell, the increase is less than would be expected and may partially be attributed to the use of the *LEU2* marker which is known to lower the copy number of 2-micron plasmids in cells [39]. Notably, the distribution of the induced population is not as clearly bimodal as in the integrated strains, with the expression level distribution being broad. We attribute this ‘noise’ in expression to the known variability of 2-micron plasmid copy numbers per cell in a population [39].

To verify that the promoters show mother-specific expression and to characterize the stringency of this, both of the strains with genomically-integrated constructs were further inspected using inverted fluorescence microscopy. To be able to track differential expression of the yeast, microscope images were captured over several hours as the yeast cells divide in a colony. These images were used to generate time-lapse movies showing colony growth and cell fluorescence. These are provided as **S1 Video** (TXC strain) and **S2 Video** (TXC2 strain).

**Fig 4:**
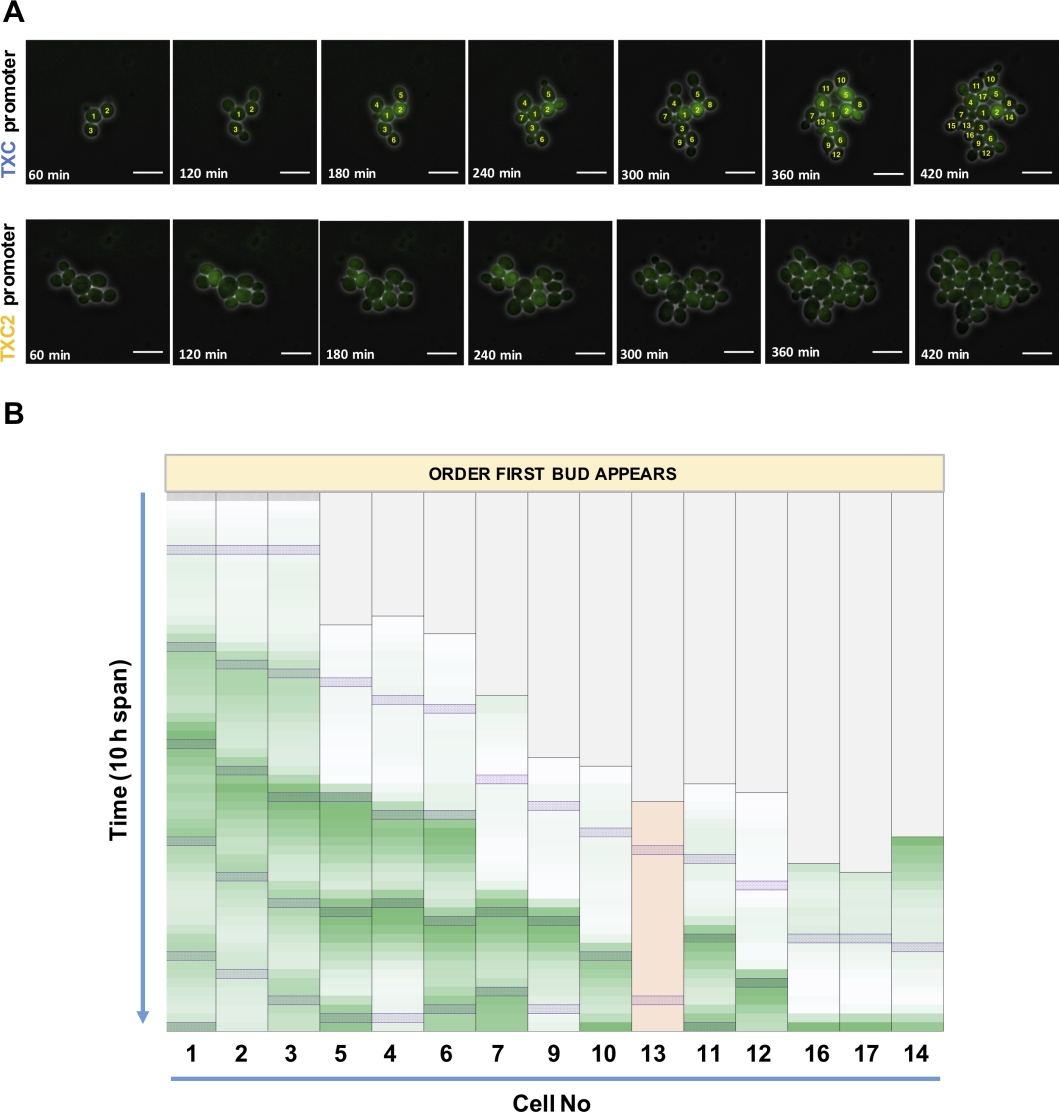
Microscopy analysis of mother-specific promoter expression. (A) Time-lapse fluorescence microscopy of the *TXC* promoter (Y02569-GPY022) and *TXC2* promoter (Y02569-GPY020) strains. Cells were grown in synthetic complete glucose media and induced with 200 ng/μl ATc for 10 h. Selected frames at 60 min intervals are shown starting 60 min after induction. Each frame is a combination of the brightfield and green fluorescence channels captured using a 60x objective. White lines represent scale bars with a length of 10 μm. (B) Time-lapse frame analysis for the *TXC* promoter strain for all cells that become mothers during the 10 h experiment. Frames representing the time when cells reach the S phase and a bud appears are designated by purple shaded boxes. The heat maps are generated based on the average fluorescence values for cells taken by ImageJ analysis and corrected for background fluorescence. Each column represents fluorescence values of one cell for each of the 60 frames taken during the 10 h span and is formatted independently of the others. Minimum fluorescence values are colored as white, maximum as green. Cells are aligned based on the order when their first daughter bud appears. Column 10 shaded in pink represents a cell that despite becoming a mother cell never fluoresces significantly above background levels. For the *TXC* strain, cells are numbered based on the order they become fully grown. Cells No 8 and No 15 are omitted from the frame analysis since they appear to arrest during the 10 hours span of the experiment thus not becoming mothers.

The Y02569-GPY022 and Y02569-GPY020 strains for *TXC* and *TXC2* promoter characterization respectively were cultured in an ONIX microfluidics platform designed for continual perfusion of growth media to cells captured in fixed positions suitable for reliable time-lapse microscopy. Growth of the strains started in glucose media without ATc and cells were then induced after 2 hours (time = 0 min) and left to grow for another 10 hours with images captured every 10 min. **Fig 4A** shows selected time-points from a time-lapse of growth for the two strains, with further images provided in **S1** and **S2 Figs**. Comparing the strength of the green signal of the fluorescence cells confirms the flow cytometry result that the *TXC* strain expresses more protein per cell than the *TXC2* strain. Most importantly, it is quite clear that at least for the *TXC* strain only a fraction of the cells in the colonies produce fluorescent signal at any given moment.

To confirm that fluorescent protein expression is indeed mother-cell specific the time-lapse microscopy images were analyzed further. As the strain with the *TXC* promoter has consistently shown the more desirable expression characteristics, this strain was the focus of the image analysis (**Fig 4B**). All frames were analyzed for their average green signal per cell (corrected for background) in order to quantify the fluorescent protein expression in each cell over time. These values were then used to generate green-scale heat maps produced for all cells in the colony that become mother cells during the 10-hour induction period. To enable easy tracking of each cell, all cells were numbered based on the order that they separate from their mother cell at the end of mitosis. A family tree of cell growth in this experiment s provided in **S3 Fig**.

Analysis of cell fluorescence in the growing colony revealed that all cells except one that become mother cells exhibit waves of green fluorescent protein expression every cell cycle, except for their first cell cycle. During this first cell cycle they are classed as daughter cells, having not produced any buds themselves and so should not be expected to express *FD-GFP*. Only one cell (No.13) appears not to follow the anticipated expression pattern and never fluoresces despite becoming a mother, perhaps due to accumulation of a mutation that inactivates the *FD-GFP* expression. Another cell (cell 14) also shows strong fluorescence at the beginning of its first cell cycle, however we attribute this to carryover of a strong *FD-GFP* signal when it divided from its mother cell No 2, rather than incorrect promoter expression. In all other cases only a faint green fluorescent signal is seen in daughter cells during their first cell cycle which we attribute to the seeping of some *FD-GFP* molecules from the mother cell cytoplasm into the bud. Furthermore, no fluorescence above background was seen in any of the cells in the colony that did not divide during the experiment, and all cells at the start of the experiment show no fluorescence, indicating that the TetR-based repression prevents leaky expression from the promoter when inducer is absent.

## Discussion

Taken together, the results of the flow cytometry **(Figs 1 and 2)** and the time-lapse microscopy **(Fig 4)** analysis of the *TXC* strain, confirm that the constructed hybrid *TXC* promoter is a mother-specific promoter that can be externally controlled by ATc induction. Upon induction, it produces a biomodal distribution of expression in the population, with only mother cells expressing the downstream reporter gene and doing so late in their second and all subsequent cell cycles. This therefore represents a successfully designed, constructed and tested hybrid promoter that gives mother-specific expression and can be externally induced. In order to ensure precise differential expression, we recommend that this promoter is used within genome-integrated constructs, as expression heterogeneity is seen when placed on a 2-micron plasmid.

A further observation from the microscopy images is that the TXC2 strain shows some fluorescence in daughter cells, but that this is less apparent for the *TXC* strain and this is likely due to *TXC2* promoter expression before cell separation at mitotic division. This is likely to be explained by the lack of the URS2 regulation site in the *TXC2* promoter, which would otherwise delay *FD-GFP* expression until after cell separation (G1 phase of the cell cycle in activated cells). Without URS2, the *HO* promoter is known to become independent of SBF control and starts production of gene transcripts upon reaching the cdc15 block stage of late anaphase [23, 27]. As a result, mother cell specificity is lost and daughter cells also transcribe the gene.

It is known that the *HO* promoter is activated only in mother cells from the second half of the G1 phase up to a point during S phase [23]. This means that for each cell the first expression from the *TXC* promoter should be expected as the cells reach the late G1 phase of their second cell cycle. In the image analysis **(Fig 4B)**, the peak of green fluorescence coincides with (or is just after) the very end of the second cell cycle as the cells move into S phase and a further bud is formed. We attribute this expression delay due to the time required to go from activating mRNA transcription from the *TXC* promoter to translating, maturing and accumulating significant levels of the green fluorescent protein per cell.

The promoter developed here offers a new alternative to the normal *HO* promoter for experiments and applications that seek to control expression between mother and daughter cells. Differential expression between mother and daughter cells has in the past been used for generating mother-rich ‘older’ populations for ageing studies, as in the previously described mother-enrichment program [40, 41]. Since the promoter we developed here is repressed in the presence of TetR, this could be used switch on and such population differences as and when are needed. It could serve the basis for generating the opposite of the mother-enrichment program and instead generating a ‘young’ daughter-rich population. This could be achieved by using the promoter to express proteins known to be toxic to yeast or halt its cell-cycle. In the absence of the ATc inducer, the population would grow as normal to a desired density, and then upon induction all cells that have produced a daughter would die or cease growth, while daughter cells would survive for a further cell cycle and bud a new cell to maintain the population. Alternatively, for synthetic biology, the promoter could be used to express any heterologous genes required for biotechnology applications that are known to significantly slow yeast growth (e.g. enzymes for natural product biosynthesis). Once inducer is applied, each cell within the culture would effectively contribute one new cell to help grow the population, before switching to slow growth and specializing in production via expression of the heterologous gene. As only around half the cells in the population would express the burdensome genes at any one time, linear growth of the population would theoretically be maintained.

In future studies, it would also be interesting to see if the concentration of inducer (ATc or doxycycline) given in the culture media could be used to tune the relative fraction of the population that is dedicated to production versus growth. Finally, because the ultimate control of expression from the *TXC* promoter is via the orthogonal TetR protein, induction or repression of its mother-specific expression can be linked to other gene regulatory systems by having the expression of the *TETR* gene change in response to environmental conditions or other inputs. Thus, mother-specific expression could be an outcome or a component of a wide variety of future genetic circuits that exploit differential expression between *S*. *cerevisiae* cells.

## Methods

### Plasmid construction

Both the pGPY022 and pGPY020 integrating plasmids carrying the *FD-GFP* gene downstream of the *TXC* and *TXC2* promoter respectively, are derived from the pTVGI plasmid (both based on pRS4D1 plasmid) provided by Dr Tom Ellis (Imperial College London) and described in Ellis *et al*. 2009 [10]. Both plasmids carry the *TETR* gene expressed from the constitutive *TEF1* promoter, have a *LEU2* selectable marker, and integrate into the *URA3* locus of the BY4741 genome via homology to a short region included in the plasmid. The *FD-GFP* gene containing a ubiquitin tag was developed and kindly provided by Dr Felix Jonas. The pGPY055 and pGPY054 2-micron plasmids were assembled using the MoClo method of modular assembly in combination with the Yeast ToolKit created by the Dueber lab [42]. All necessary promoter, open reading frame (ORF) and terminator parts were first amplified by PCR and stored in ‘part-level plasmids’ as required for the YTK system. They were then assembled into 2-micron plasmids so that the *FD-GFP* sequence is downstream of either the *TXC* or *TXC2* hybrid promoter. Plasmids were constructed to also include a *LEU2* marker and the *TETR* gene with *TEF1* promoter. Annotated sequences for the promoters used in this study and for *FD-GFP* are provided in the **S1 Text** along with plasmid maps (**S1 Fig**). Plasmids from this study will be made available via Addgene.org.

### Strains and cultures

All engineered strains were derived from the haploid *S*. *cerevisiae* Y02569 (BY4741; MATa; ura3Δ0; leu2Δ0; his3Δ1; met15Δ0; YJR092w::kanMX4) strain provided by EUROSCARF. Cells are cultured in synthetic complete drop-out glucose media (SC-Glu) for transformation and general proliferation and in synthetic complete drop-out glucose media (SC-Glu) media with 200 ng/μl ATc for hybrid promoter induction. During the time-lapse and ONIX experiments, cells were grown at 30°C inside the microscope. For all other cultures, cells were grown in liquid cultures at 30°C with shaking at 225 rpm.

### Inverted microscope image capture

Images were taken through a 60x CPI60 objective mounted on a Nikon Eclipse Ti inverted microscope with live cells imaged using the CellASIC ONIX Microfluidic platform (Merck Millipore). Cells were grown in Y04C-02-5PK plates and flow rate was adjusted at Psi = 4 which was determined to be good for yeast cells. To visualize the samples a Phase filter 3 is used to enhance contrast and Brightfield illumination. For fluorescence capture, excitation, emission filters, and exposures were respectively 480 nm, 535 nm, 1000 ms for the GFP channel. During time-lapse experiments, the software autofocus function of the microscope is used to adjust for any potential movement of the cells during growth in order to keep clear track of the samples. NIS-Elements Microscope Imaging Software (Nikon) is used for capturing and ImageJ (National Institutes of Health) is used for image presentation.

### Flow cytometry analysis

Flow cytometry assays were performed using the Attune NxT flow cytometer with the Attune NxT autosampler attachment from ThermoFisher Scientific. A 488 nm laser was used for excitation of green fluorescence detecting through a 530 nm band-pass filter (BL1). The voltages of the FSC, SSC and BL1 channels for the promoter characterization experiments were 200, 320 and 480 respectively. A threshold of 3.0 x 10^3^ A.U. was applied to the forward (FSC) scatter to minimize non-yeast events. Data analysis was performed using FlowJo software (Tree Star), gating samples for forward scatter and side scatter to exclude non-yeast events and obtaining fluorescence values from BL1-H (height) channels.

## Supporting information

S1 Text Annotated promoter and *FD-GFP* part sequences. (DOCX)

S1 Fig Simplified plasmid maps of the integrating pGPY020 and pGPY022 and the episomal pGPY054 and pGPY055 plasmids. All plasmids carry the *tetR* gene under the TEF1 promoter, the leucine (*LEU2)* yeast selection marker, a bacterial ColE1 origin and either the ampicillin (*ampR*) or the kanamycin (kanR)resistance marker. Genes are represented by coloured arrow-shaped boxes and key promoters by grey boxes upstream of the genes. Plasmid sizes are given in base pairs (bp).
(TIFF)

S2 Fig Time-lapse fluorescence microscopy of the Y02569-GPY022 strain. Cells are grown in synthetic complete glucose media and induced with 200 ng/μl ATc for 10 h. The first frame corresponds to the moment when induction started and after that, frames at 30 minute intervals are shown. Both brightfield and green fluorescence images were captured using a 60x objective. Brightfield images are captured using a Phase 3 contrast filter. Each frame is a combination of the brightfield and green fluorescence channels. On each frame, cells are numbered based on the moment they appear to separate from the mother cell (end of mitosis). White lines represent scale bars with a length of 10 nm.
(TIFF)

S3 Fig Time-lapse fluorescence microscopy of the Y02569-GPY020 strain. Cells are grown in synthetic complete glucose media and induced with 200 ng/μl ATc for 10 h. The first frame corresponds to the moment when induction started and after that, frames at 30 minute intervals are shown. White arrows are pointing to some of the daughter cells that exhibit fluorescence due to leakage from the mother cells. Both brightfield and green fluorescence images were captured using a 60x objective. Brightfield images are captured using a Phase 3 contrast filter. Each frame is a combination of the brightfield and green fluorescence channels. White lines represent scale bars with a length of 10 nm.
(TIFF)

S4 Fig Family trees of the cells shown in the time-lapse experiment of the Y02569-GPY022 strain. One tree for each one of the starting cells was created. Numbers in parenthesis shown in green represent the frame numbers when the cells are about to separate from the mother cells (end of mitosis). Numbers in parenthesis shown in orange represent the frame numbers when the cells appear as buds since they don’t achieve separation before the end of the experiment.
(TIFF)

S1 Video Time-lapse fluorescence microscopy of the Y02569-GPY022 strain. (AVI)

S2 Video Time-lapse fluorescence microscopy of the Y02569-GPY020 strain. (AVI)
