# Supplementary material for: Construction of hybrid regulated mother-specific yeast promoters for inducible differential gene expression
Source: PLoS One. 2018 Mar 22;13(3):e0194588. doi: 10.1371/journal.pone.0194588 (PMC5864024; doi:10.1371/journal.pone.0194588)
Supplement: S1 Text — (DOCX) [file pone.0194588.s001.docx]

**Supplementary material**

Construction of hybrid regulated mother-specific yeast promoters for inducible differential gene expression

Georgios Pothoulakis^1,2^ and Tom Ellis^1,2*^

^1^Centre for Synthetic Biology and Innovation, Imperial College London, London, United Kingdom

^2^Department of Bioengineering, Imperial College London, London, United Kingdom

*Corresponding author

E-mail: [t.ellis@imperial.ac.uk](mailto:t.ellis@imperial.ac.uk) (TE)

**Part Sequences**

**TX promoter sequence**

**GAL1 UAS**

GGAATTCGAAGTACGGATTAGAAGCCGCCGAGCGGGTGACAGCCCTCCGAAGGAAGACTCTCCTCCGTGCGTCCTCGTCTTCACCGGTCGCGTTCCTGAAACGCAGATGTGCCTCGCGCCGCACTGCTCCGAACAATAAAGATTCTACAATACTAGCTTTTATGGTTATGAAGAGGAAAAATTGGCAGTAACCTGGCCCCACAAACCTTCAAATGAACGAATCAAATTAACAACCATAGGATGATAATGCGATTAGTTTTTTAGCCTTATTTCTGGGGTACTGCAGCAGCGAAGCGATGATTTTTGATCTATTAACAGATATATAAATGCAAAAACTGTCCCTATCAGTGATAGAGATCTCCCTATCAGTGATAGAGATATTACTTCTTATTCAAATGTAATAAAAGTATCAACAAAAAATTGTTAATATACCTCTATACTTTAACGTCAAGGAGAAAAAACCCCGGATCCTATTAAA

**tetO**

**tetO**

**TATA**

**HO promoter sequence**

CATTTTTGTTTCTTTTGGACAAATGTTGTTTGCATTTATGATCCGTTATATTTTGATCTAATGTAGAGTTGCACGTAGTTCTTACTGGCAAAGAAATCGATGCATACCAAAAAAGAATAAAGGTGATATTTGATCTTTACCGTTTAGTTCCAACGTAAAATTGTGCCTTTGGACTTAAAATGGCGTGGCAGAACTAACTCTTTATTTTTCCAAATCAGAAAAATTAATATGTTTTGCCGCGTTAAAACCTACATCAAAAAAGGCGGATCAAGATGTATGAAAGAAAGTGCGTAGAATAACGAACATTCATAGCTGTTCTGGAGGCTTTACAAAAGGTAATCTTTGTTAGGTGCGATTTTATCCGAAAAGCAATTACTCTCTATGTTAGTCATACAAACTGACTTCTGTGAGAGAATTATTTTCATATCAACGTAAGATCACATGGTTCCTTTATCAAGTACTACTATCATTCCATTATATGACCTATTTACTTCTTGAATCTTAGAGCTCATAATTCAAGCAAGTTGCGGAGCTAAGAATTTCACATGTTGTTGAACTTAACAATCTTCATTATACCCAATCGCTGCGTGCTGGAATTATGTTAAAAGTTACATCCTTTTTTTCATTTTTCCCTACGCTCAGGGCACTGTACTGCCCGTGCCTGCGATGAGATACATCAATTTAAAAAAAAAACCAGCATGCTATAATGCTGGAGCAAAAATTTCAATCAGAAATAGAAAAGACCTCAACAGTAATTAACCCAAAGGGGTATCAAATAATCGATGTGCTTTTTCACTCTACGAATGATCTGTGAGAAACTGATTTGGGCCGAATCGCGTAAAAAGTTTGATTCGTGGCGGCTAATGTCTGAGGGGCTCCAACAGGCTCGTAGAGCCTCGTTTCTTGAGGGCACAAAATGTCCAGGTAATATTCCCAAGAAAGAACCGCAGAGTGCTTTGATAAATCGGTTACAGGTCTTAACGTAGGTTTTGTCTCGCTAATTGCTATTGAGTAAGTTCGATCCGTTTGGCGTCTTTTGGGGTGTAACGCCAAACTTATTACTTTTCCTATTTGAGGTTGGTATTGATTGTTGTCAAAGAATGAAAATATACACAAACGCCACAATATACGTACCAGGTTCACGAAAACTGATCGTATGGTTCATACCCTGACTTGGCAAACCTAATGTGACCGTCGCTGATTAGCGGATCACGAAAAGTGATCTCGATACAATTAGAGGATCCACGAAAATGATGTGAATGAATACATGAAAGATTCATGAGATCTGACAACATGGTAGACGTGTGTGTCTCATGGAAATTGATGCAGTTGAAGACATGTGCGTCACGAAAAAAGAAATCAATCCTACACAGGGCTTAAGGGCAAATGTATTCATGTGTGTCACGAAAAGTGATGTAACTAAATACACGATTACCATGGAAATTAACGTACCTTTTTTGTGCGTGTATTGAAATATTATGACATATTACAGAAAGGGTTCGCAAGTCCTGTTTCTATGCCTTTCTCTTAGTAATTCACGAAATAAACCTATGGTTTACGAAATGATCCACGAAAATCATGTTATTATTTACATCAACATATCGCGAAAATTCATGTCATGTCCACATTAACATCATTGCAGAGCAACAATTCATTTTCATAGAGAAATTTGCTACTATCACCCACTAGTACTACCATTGGTACCTACTACTTTGAATTGTACTACCGCTGGGCGTTATTAGGTGTGAAACCACGAAAAGTTCACCATAACTTCGAATAAAGTCGCGGAAAAAAGTAAACAGCTATTGCTACTCAAATGAGGTTTGCAGAAGCTTGTTGAAGCATGATGAAGCGTTCTAAACGCACTATTCATCATTAAATATTTAAAGCTCATAAAATTGTATTCAATTCCTATTCTAAATGGCTTTTATTTCTATTACAACTATTAGCTCTAAATCCATATCCTCATAAGCAGCAATCAATTCTATCTATACTTTAAA

**URS1**

**URS2**

**TXC promoter sequence**

**URS1**

TTTTGATCTAATGTAGAGTTGCACGTAGTTCTTACTGGCAAAGAAATCGATGCATACCAAAAAAGAATAAAGGTGATATTTGATCTTTACCGTTTAGTTCCAACGTAAAATTGTGCCTTTGGACTTAAAATGGCGTGGCAGAACTAACTCTTTATTTTTCCAAATCAGAAAAATTAATATGTTTTGCCGCGTTAAAACCTACATCAAAAAAGGCGGATCAAGATGTATGAAAGAAAGTGCGTAGAATAACGAACATTCATAGCTGTTCTGGAGGCTTTACAAAAGGTAATCTTTGTTAGGTGCGATTTTATCCGAAAAGCAATTACTCTCTATGTTAGTCATACAAACTGACTTCTGTGAGAGAATTATTTTCATATCAACGTAAGATCACATGGTTCCTTTATCAAGTACTACTATCATTCCATTATATGACCTATTTACTTCTTGAATCTTAGAGCTCATAATTCAAGCAAGTTGCGGAGCTAAGAATTTCACATGTTGTTGAACTTAACAATCTTCATTATACCCAATCGCTGCGTGCTGGAATTATGTTAAAAGTTACATCCTTTTTTTCATTTTTCCCTACGCTCAGGGCACTGTACTGCCCGTGCCTGCGATGAGATACATCAATTTAAAAAAAAAACCAGCATGCTATAATGCTGGAGCAAAAATTTCAATCAGAAATAGAAAAGACCTCAACAGTAATTAACCCAAAGGGGTATCAAATAATCGATGTGCTTTTTCACTCTACGAATGATCTGTGAGAAACTGATTTGGGCCGAATCGCGTAAAAAGTTTGATTCGTGGCGGCTAATGTCTGAGGGGCTCCAACAGGCTCGTAGAGCCTCGTTTCTTGAGGGCACAAAATGTCCAGGTAATATTCCCAAGAAAGAACCGCAGAGTGCTTTGATAAATCGGTTACAGGTCTTAACGTAGGTTTTGTCTCGCTAATTGCTATTGAGTAAGTTCGATCCGTTTGGCGTCTTTTGGGGTGTAACGCCAAACTTATTACTTTTCCTATTTGAGGTTGGTATTGATTGTTGTCAAAGAATGAAAATATACACAAACGCCACAATATACGTACCAGGTTCACGAAAACTGATCGTATGGTTCATACCCTGACTTGGCAAACCTAATGTGACCGTCGCTGATTAGCGGATCACGAAAAGTGATCTCGATACAATTAGAGGATCCACGAAAATGATGTGAATGAATACATGAAAGATTCATGAGATCTGACAACATGGTAGACGTGTGTGTCTCATGGAAATTGATGCAGTTGAAGACATGTGCGTCACGAAAAAAGAAATCAATCCTACACAGGGCTTAAGGGCAAATGTATTCATGTGTGTCACGAAAAGTGATGTAACTAAATACACGATTACCATGGAAATTAACGTACCTTTTTTGTGCGTGTATTGAAATATTATGACATATTACAGAAAGGGTTCGCAAGTCCTGTTTCTATGCCTTTCTCTTAGTAATTCACGAAATAAACCTATGGTTTACGAAATGATCCACGAAAATCATGTTATTATTTACATCAACATATCGCGAAAATTCATGTCATGTCCACATTAACATCATTGCAGAGCAACAATTCATTTTCATAGAGAAATTTGCTACTATCACCCACTAGTACTACCATTGGTACCTACTACTTTGAATTGTACTACCGCTGGGCGTTATTAGGTGTGAAACCACGAAAAGTTCACCATAACTTCGAATAAAGTCGCGGAAAAAAGTAAACAGCTATTGCTACTCAAATGAGGTTTGCAGAAGCTTGTTGAAGCATGATGAAGCGTCTGCAGCAGCGAAGCGATGATTTTTGATCTATTAACAGATATATAAATGCAAAAACTGTCCCTATCAGTGATAGAGATCTCCCTATCAGTGATAGAGATATTACTTCTTATTCAAATGTAATAAAAGTATCAACAAAAAATTGTTAATATACCTCTATACTTTAACGTCAAGGAGAAAAACCCCGGATCCTATTAAA

**URS2**

**tetO**

**tetO**

**TATA**

**TXC2 promoter sequence**

**URS1**

TTTTGATCTAATGTAGAGTTGCACGTAGTTCTTACTGGCAAAGAAATCGATGCATACCAAAAAAGAATAAAGGTGATATTTGATCTTTACCGTTTAGTTCCAACGTAAAATTGTGCCTTTGGACTTAAAATGGCGTGGCAGAACTAACTCTTTATTTTTCCAAATCAGAAAAATTAATATGTTTTGCCGCGTTAAAACCTACATCAAAAAAGGCGGATCAAGATGTATGAAAGAAAGTGCGTAGAATAACGAACATTCATAGCTGTTCTGGAGGCTTTACAAAAGGTAATCTTTGTTAGGTGCGATTTTATCCGAAAAGCAATTACTCTCTATGTTAGTCATACAAACTGACTTCTGTGAGAGAATTATTTTCATATCAACGTAAGATCACATGGTTCCTTTATCAAGTACTACTATCATTCCATTATATGACCTATTTACTTCTTGAATCTTAGAGCTCATAATTCAAGCAAGTTGCGGAGCTAAGAATTTCACATGTTGTTGAACTTAACAATCTTCATTATACCCAATCGCTGCGTGCTGGAATTATGTTAAAAGTTACATCCTTTTTTTCATTTTTCCCTACGCTCAGGGCACTGTACTGCCCGTGCCTGCGATGAGATACATCAATTTAAAAAAAAAACCAGCATGCTATAATGCTGGAGCAAAAATTTCAATCAGAAATAGAAAAGACCTCAACAGTAATTAACCCAAAGGGGTATCAAATAATCGATGTGCTTTTTCACTCTACGAATGATCTGTGAGAAACTGATTTGGGCCGAATCGCGTAAAAAGTTTGATTCGTGGCGGCTAATGTCTGAGGGGCTCCAACAGGCTCGTAGAGCCTCGTTTCTTGAGGGCACAAAATGTCCAGGTAATATTCCCAAGAAAGAACCGCAGAGTGCTTTGATAAATCGGTTACAGGTCTTAACGTAGGTTTTGTCTCGCTACTGCAGCAGCGAAGCGATGATTTTTGATCTATTAACAGATATATAAATGCAAAAACTGTCCCTATCAGTGATAGAGATCTCCCTATCAGTGATAGAGATATTACTTCTTATTCAAATGTAATAAAAGTATCAACAAAAAATTGTTAATATACCTCTATACTTTAACGTCAAGGAGAAAAACCCCGGATCCTATTAAA

**tetO**

**TATA**

**tetO**

**FD-GFP sequence**

**Ubiquitin recognition**

ATGCAGATTTTCGTCAAGACTTTGACCGGTAAAACCATAACATTGGAAGTTGAATCTTCCGATACCATCGACAACGTTAAGTCGAAAATTCAAGACAAGGAAGGTATCCCTCCAGATCAACAAAGATTGATCTTTGCCGGTAAGCAGCTAGAAGACGGTAGAACGCTGTCTGATTACAACATTCAGAAGGAGTCCACCTTACATCTTGTGCTAAGGCTAAGAGGTGGTGATGGGAAACTTGGTCGACAAGATCCACCTGTCGCCACCATGGTTTCCAAGGGTGAAGAGCTATTTACTGGGGTTGTACCCATTTTGGTAGAACTGGACGGAGATGTAAACGGACATAAATTCTCTGTTAGAGGTGAGGGCGAAGGCGATGCCACCAATGGTAAATTGACTCTGAAGTTTATATGCACTACGGGTAAATTACCTGTTCCTTGGCCAACCCTAGTAACAACTTTGACATATGGTGTTCAATGTTTCTCAAGATACCCAGACCATATGAAAAGGCATGATTTCTTTAAAAGTGCTATGCCAGAAGGCTACGTGCAAGAGAGAACTATCTCCTTTAAGGATGACGGTACGTATAAAACACGAGCAGAAGTGAAATTCGAAGGGGATACACTAGTTAATCGCATCGAATTAAAGGGTATAGACTTTAAGGAAGATGGTAATATTCTCGGCCATAAACTTGAGTATAATTTCAACTCGCATAATGTGTACATTACAGCTGACAAACAAAAGAACGGAATTAAAGCGAATTTTAAAATCAGGCACAACGTCGAAGATGGGTCTGTTCAACTTGCCGATCATTATCAGCAAAACACCCCTATTGGTGATGGTCCAGTCTTGTTACCCGATAATCACTACTTAAGCACACAGTCTAGATTGTCAAAAGATCCGAATGAAAAGCGTGATCACATGGTTTTATTGGAATTTGTCACCGCTGCAGGAATAACTCACGGAATGGACGAGCTTTATAAGGGATCCTAA

**GFP**
