# Supplementary figures and images for: Construction of hybrid regulated mother-specific yeast promoters for inducible differential gene expression
Source: PLoS One. 2018 Mar 22;13(3):e0194588. doi: 10.1371/journal.pone.0194588 (PMC5864024; doi:10.1371/journal.pone.0194588)

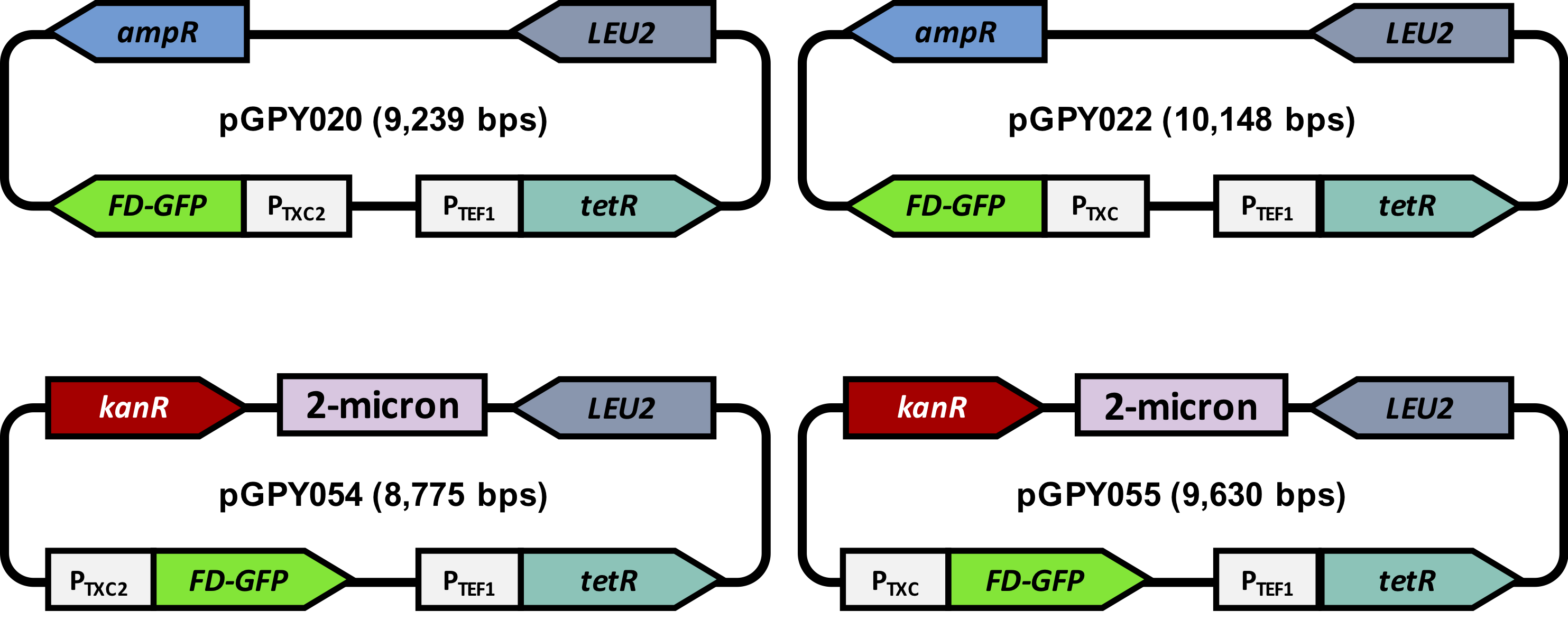

Supplement: S1 Fig — All plasmids carry the tetR gene under the TEF1 promoter, the leucine (LEU2) yeast selection marker, a bacterial ColE1 origin and either the ampicillin (ampR) or the kanamycin (kanR)resistance marker. Genes are represented by coloured arrow-shaped boxes and key promoters by grey boxes upstream of the genes. Plasmid sizes are given in base pairs (bp). (TIFF) [file pone.0194588.s002.tiff]

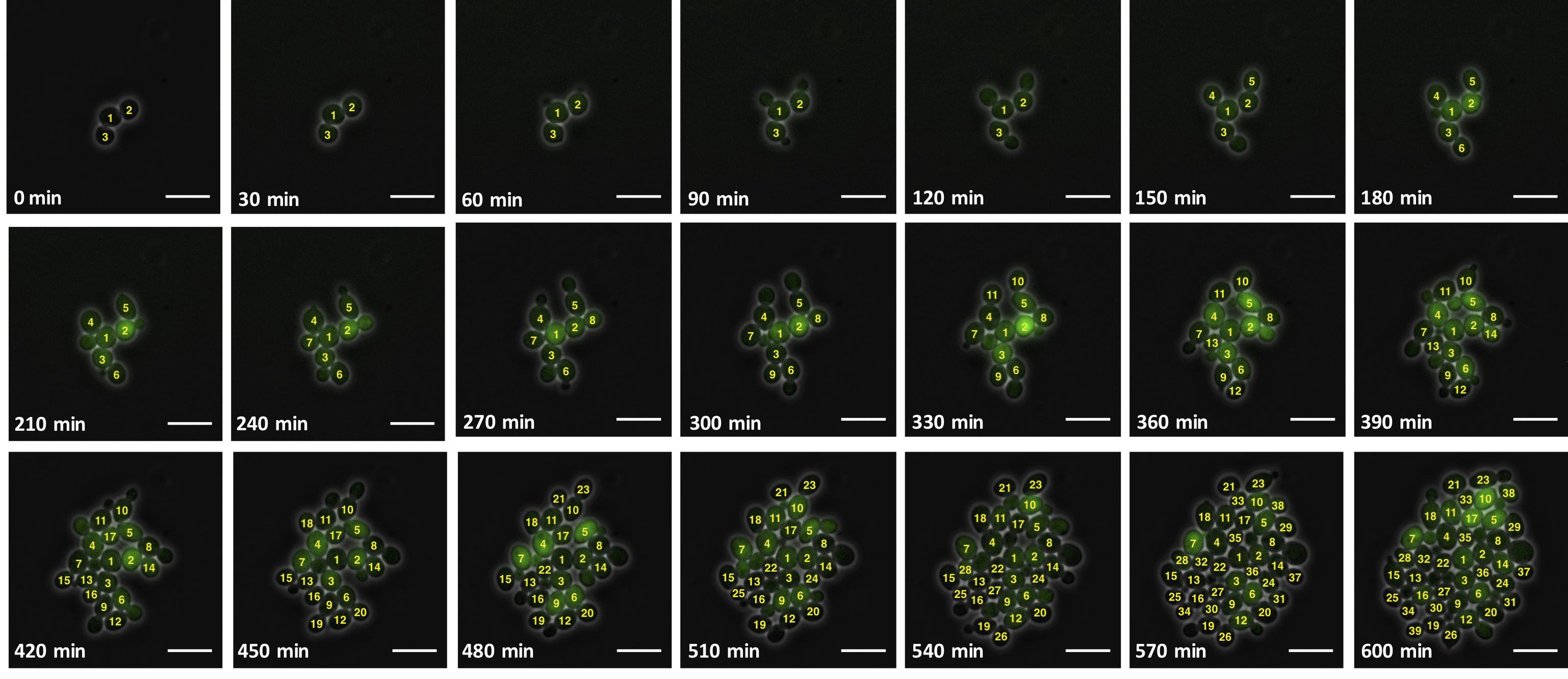

Supplement: S2 Fig — Cells are grown in synthetic complete glucose media and induced with 200 ng/μl ATc for 10 h. The first frame corresponds to the moment when induction started and after that, frames at 30 minute intervals are shown. Both brightfield and green fluorescence images were captured using a 60x objective. Brightfield images are captured using a Phase 3 contrast filter. Each frame is a combination of the brightfield and green fluorescence channels. On each frame, cells are numbered based on the moment they appear to separate from the mother cell (end of mitosis). White lines represent scale bars with a length of 10 nm. (TIFF) [file pone.0194588.s003.tiff]

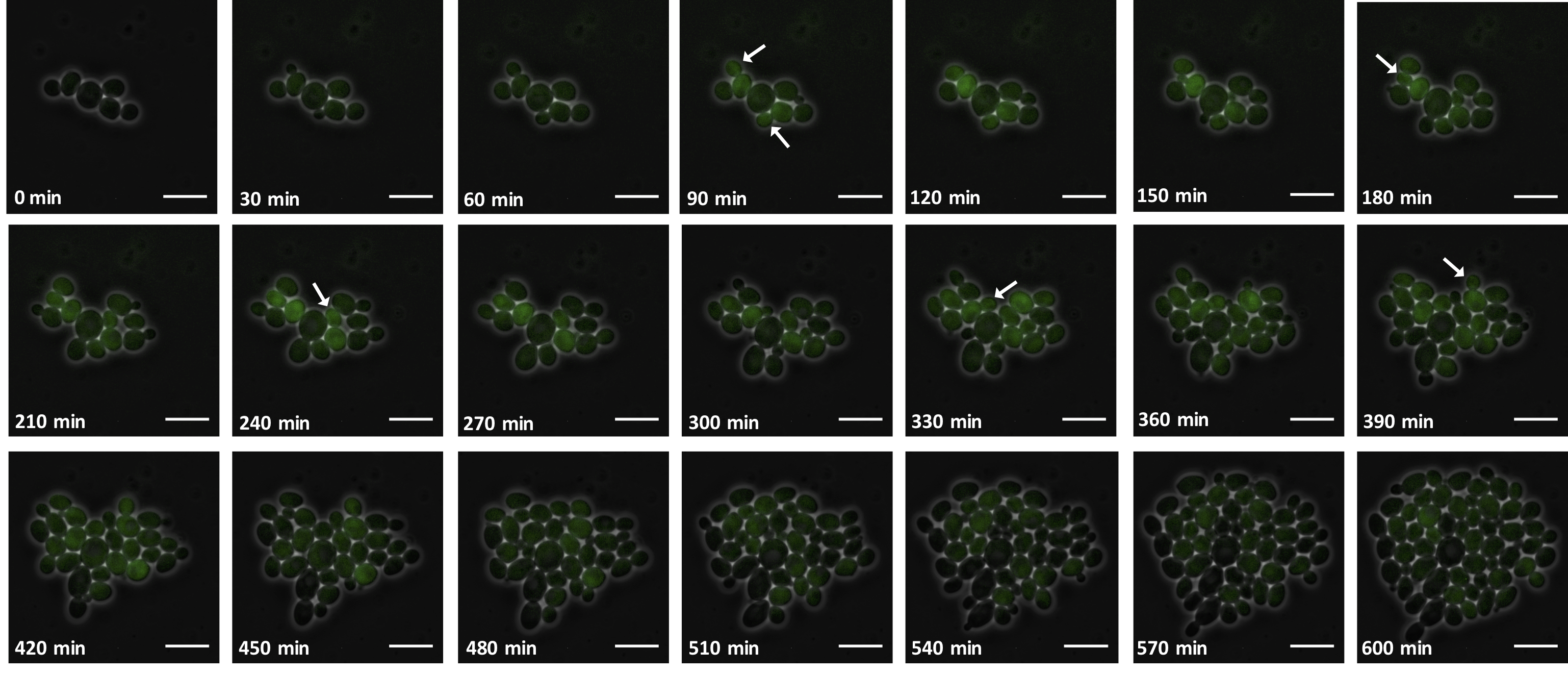

Supplement: S3 Fig — Cells are grown in synthetic complete glucose media and induced with 200 ng/μl ATc for 10 h. The first frame corresponds to the moment when induction started and after that, frames at 30 minute intervals are shown. White arrows are pointing to some of the daughter cells that exhibit fluorescence due to leakage from the mother cells. Both brightfield and green fluorescence images were captured using a 60x objective. Brightfield images are captured using a Phase 3 contrast filter. Each frame is a combination of the brightfield and green fluorescence channels. White lines represent scale bars with a length of 10 nm. (TIFF) [file pone.0194588.s004.tiff]

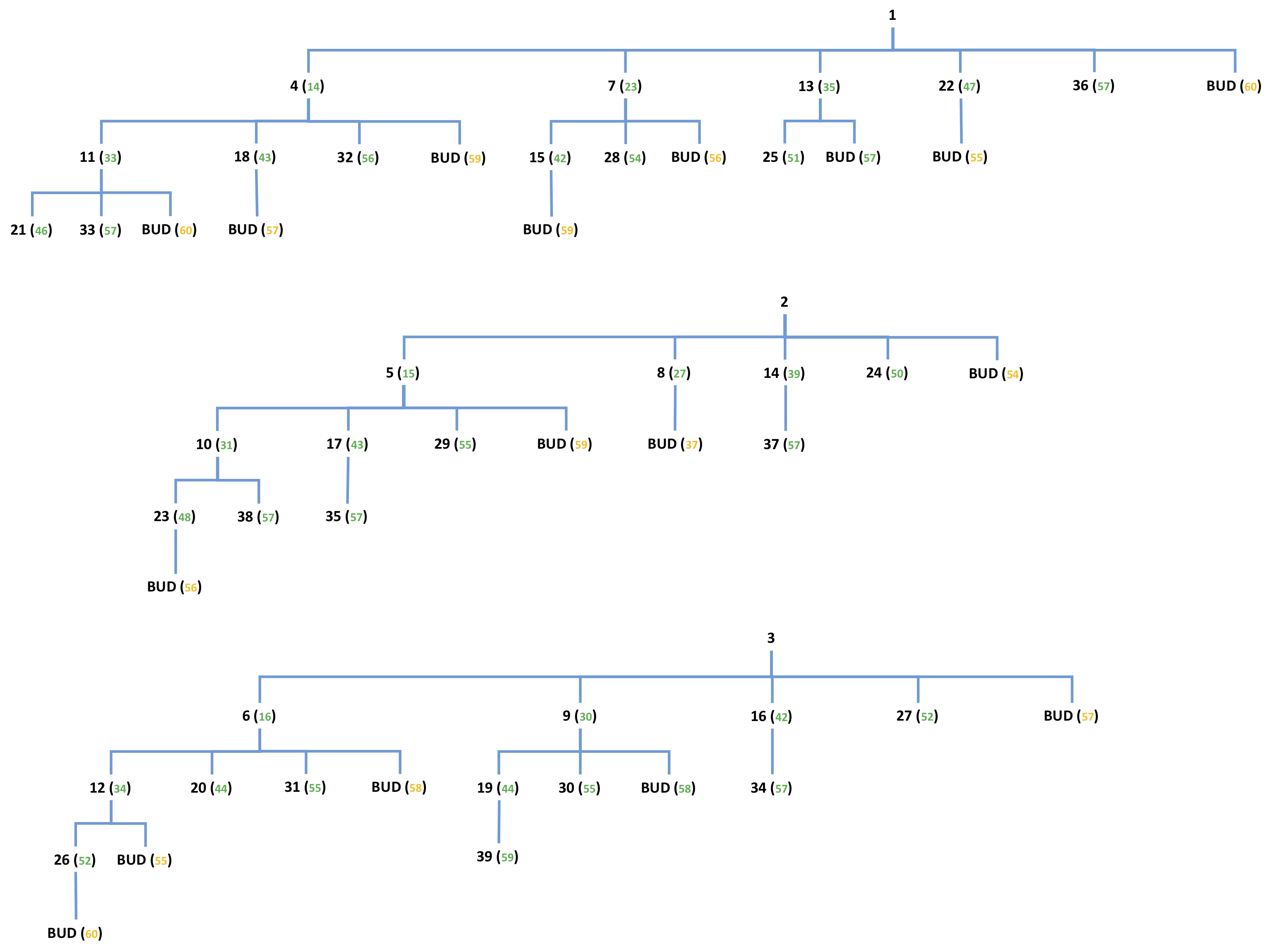

Supplement: S4 Fig — One tree for each one of the starting cells was created. Numbers in parenthesis shown in green represent the frame numbers when the cells are about to separate from the mother cells (end of mitosis). Numbers in parenthesis shown in orange represent the frame numbers when the cells appear as buds since they don’t achieve separation before the end of the experiment. (TIFF) [file pone.0194588.s005.tiff]
